# Radiation-Induced Lumbosacral Plexopathy

**DOI:** 10.7759/cureus.36842

**Published:** 2023-03-29

**Authors:** Luís R Almeida, Diogo Faustino, Luciano R Esteves, Cristiano Gante, Adriana W Soares, Tiago Oliveira, João L Dias, Luis Dias

**Affiliations:** 1 Internal Medicine, Centro Hospitalar Universitário Lisboa Central - Hospital de São José, Lisbon, PRT; 2 Neurology, Centro Hospitalar Universitário Lisboa Central - Hospital de São José, Lisbon, PRT; 3 Radiology, Centro Hospitalar Universitário Lisboa Central - Hospital de São José, Lisbon, PRT

**Keywords:** paraparesis, radiation-induced lesion, radiotherapy toxicity, myokymia, lumbosacral plexopathy

## Abstract

Lumbosacral plexopathy (LSP) encompasses a group of disorders affecting post-ganglionic fibers derived from the L1-S4 roots. The differential diagnosis is challenging and includes other neuropathies of medullary, radicular, or peripheral origin. Defining the etiology is equally crucial, as LSP management relies on its cause. A thorough clinical history should address potential neoplastic disease (new-onset, progression, or relapse), diabetes mellitus, lumbar or pelvic trauma, and previous exposure to radiation. This is the case of a 78-year-old male, with a history of prostatic adenocarcinoma, treated with image-guided radiation therapy and hormone therapy five years before, with no evidence of relapse on follow-up. The patient presented with bilateral weakness, numbness, and paresthesia of lower limbs, gradually progressing over a three-month period, and followed by an acute worsening with inability to stand or walk. He also referred to distal mild edema, episodic hematuria, and urinary incontinence. Physical examination revealed paraparesis affecting proximal and distal leg muscles, along with bilateral hypoesthesia, impaired deep tendon reflexes, and proprioception below knee level. Pelvic, dorsal, and lumbosacral MRI excluded neoplastic lesions but identified somatic fracture of L5 without medullary or conus medullaris compromise. These findings did not explain the clinical picture. Further neurophysiologic studies characterized sensory-motor deficits as post-ganglionic, with specific spontaneous discharges of the muscle fibers, known as myokymia. These findings were consistent with radiation-induced LSP and were supported by MRI. Radiation-induced cystitis was also documented in pelvic MRI and urethral cystoscopy. This case highlights the clinical picture and differential diagnosis of radiation-induced LSP. Despite more typical symptoms and course, a neoplastic origin should always be carefully investigated and excluded. Radiation protocol should be carefully accessed, and its complications should not be overlooked, as they might cause severe morbidity.

## Introduction

Plexopathy is a general term to describe the conditions affecting brachial or lumbosacral plexuses. The lumbosacral plexus comprises post-ganglionic fibers derived from the anterior rami of L1-S4 roots, with variable contribution of T12 roots [1,2]. Lumbosacral plexus injury is often diffuse and may have simultaneous involvement of roots and peripheral nerves, blurring the neurological examination and the definition of the precise level of the lesion on a clinical basis [1,3].

There is a myriad of etiologies for lumbosacral plexopathy (LSP), including diabetes mellitus, neoplasia, trauma, post-surgery, obstetric, systemic or local infection, vascular inflammation, inflammatory or granulomatous diseases, and post-radiation therapy [1,3,4]. The most common etiologies are diabetic and idiopathic radiculoplexopathy [5,6]. Diabetic amyotrophy has a particular incidence in individuals with type 2 diabetes mellitus and adequate glycemic control. It presents typically with acute asymmetric pain, followed by weakness and autonomic impairment. Idiopathic radiculoplexopathy is mainly indistinctive from the latter but occurs in non-diabetic patients [5,6].

Neoplastic LSP typically occurs due to direct locoregional invasion from the surrounding structures, including abdominal or pelvic organs, as well as arising metastasis [7,8]. Specific perineural invasion is less frequent, but lymphomas and adenocarcinomas may have a confined longitudinal spread across the neural fibers (e.g., vagina, cervix, rectum, prostate) [1,7,9,10]. Other sources include meningeal and hematogenous dissemination, paraneoplastic syndromes, and bone and muscular malignancies [1,10]. Pain is the major symptom associated with neoplastic LSP [1,4,8]. More commonly, pain is described as localized and unilateral, with mixed mechanic and radicular pattern, but it may present as a bilateral and radicular syndrome, or rarely with no pain [6,10]. Neurological deficits may be evident at presentation or develop gradually in the disease course, including weakness, sensory loss, and paresthesia [1,6,8,10]. Paraneoplastic neurological syndromes with LSP are rare, but cases of isolated myelopathy or mixed neuropathies are part of its differential. These neuroimmune clusters are more often presented with brainstem findings, superior function, or neuropsychiatric impairment [11]. Isolated lower motor neuron syndrome is rare. The latter was more associated with breast and small-cell lung cancer, and certain antineuronal antibodies such as anti-Hu, anti-CRMP5, or antiamphiphysin [11].

Radiation-induced plexopathy is more often identified in the cervical plexus, after radiation therapy for breast or lung cancer [4,12]. Radiation-induced lumbosacral plexopathy (RILP) may arise from radiation therapy directed to any pelvic organ [3,4]. It is less frequent, but the incidence has been increasing with improved long-term cancer survival [13]. The mechanisms of radiation neurotoxicity and RILP have been highly debated, including phenomena of chronic inflammation, microvascular injury, and fibrosis, and they may progress for several years before being clinically apparent [1,12-14]. Case reports describe symptoms of RILP up to 36 years before radiotherapy, with five year median onset time [12-16]. Presentation is insidious and gradual, and there can be periods of acute worsening of symptoms [1,8]. Motor deficit presents like a typical lower motor neuron syndrome with proximal and/or distal weakness of the limb, and muscular atrophy. The deficits can be bilateral or unilateral. Sensory and proprioceptive impairment can occur simultaneously or be delayed in time, resembling sensory-motor polyneuropathy. Pain can be present in a minority of patients with a neuropathic pattern. The potential to develop RILP is dependent on many factors, such as total radiation dose, dose per fraction, volume of irradiated tissue, applied technique, hot spot high dosing (field junctions), concomitant use of taxane and platinum chemotherapy, and other predisposing factors like advanced age, obesity, hypertension, dyslipidemia, diabetes and previous surgery within the irradiated area [5,13,16,17]. External beam radiation is less prone to RILP than intracavitary or intraoperative procedures [1,12,13]. Intensity-modulated (IMRT) and image-guided radiation therapy (IGRT) can minimize the exposition of surrounding healthy tissues, with more precise distribution of radiation, as well as high fractionated doses (2-2.5 Gy/cycle/day) [12,13,16,18]. There are no consensual and specific cut-off values for RILP, but total radiation dose >50 Gy on lumbosacral plexus is thought to increase the risk [13,16]. Moreover, total dose of 81 Gy is also significantly associated with more risk of genitourinary and gastrointestinal toxicity compared to 66 Gy dose [18]. Many factors are to be considered, including the best risk-benefit balance for the patient. Long-term studies are needed to better evaluate long-term radiation toxicity [18].

The study of LSP includes electrophysiologic studies, imaging methods, and laboratory workup. Electromyography and evoked potentials testing are warranted to characterize the severity of the deficits and define their level and extension [6,7]. Spontaneous consecutive discharges with slow irregular firing frequencies, known as myokymia are very specific for RILP, but they do not exclude concomitant causes [1,6,17]. Investigation should include pelvic, lumbar, and sacral MRI with enhanced contrast to assess the neuraxis and radicular roots and exclude other concurrent causes [13,16,19,20]. PET scan with fluorodeoxyglucose enhancement can be used as an alternative to MRI, or if its findings were inconclusive [8,12,19].

## Case presentation

We present a case of a 78-year-old man, previously independent in activities of daily living. He had a known history of arterial hypertension, obesity, chronic obstructive pulmonary disease, atrial fibrillation, and cerebrovascular disease due to cardioembolic ischemic stroke under anticoagulation therapy, and prostatic adenocarcinoma (Gleason 8 - 4+4; cT2bN0M0). The tumor was treated five years before, with onboard imaging IGRT, high fractionated doses of 2 Gy/cycle/day (40 sessions for two months), and total dose of 50 Gy for pelvic irradiation, 64 Gy in the seminal vesicles, and 80 Gy for prostate gland irradiation. The patient also completed androgen blockade hormone therapy (goserelin and cyproterone) for one year, with no clinical, biochemical, or imaging evidence of relapse on follow-up surveillance.

He was admitted to the ward with complaints of symmetrical weakness, numbness, and mild edema of the lower limbs ongoing for three months. The symptoms had been progressing gradually, with two fall episodes in the latter month, and sudden worsening, with inability to stand or walk in the previous four days. No back or limb pain was reported. The patient referred to previous episodes of macroscopic hematuria, which were confirmed by urinalysis on two occasions in the ward. Physical examination identified lower limb paraparesis, including the hip abductors, adductors, and distal muscle groups (Medical Research Council grade 4), distal leg hypoesthesia involving pain and temperature sensation, with abolished lower limb proprioception, and deep tendon reflexes (patellar and Achilles). The patient presented total inability to stand without support. Hyponatremia was noted on laboratory workup, along with low serum osmolality, low serum uric acid, upper-normal urinary osmolality, and urinary sodium. Table 1 summarizes the laboratory findings.

**Table 1:** Laboratory investigation. L: low; UN: upper normal

Variables	Results	Reference range
Serum sodium	124 (L)	136-145 mmol/L
Serum osmolality	254 (L)	275-300 mOsmol/kg
Serum uric acid	3.4 (L)	4.0-7.0 mg/dL
Serum albumin	25 (L)	32.0-46.0 g/L
Urinary sodium	58 (UN)	(Variable) mmol/L
Urinary osmolality	373 (UN)	(Variable) mOsmol/kg

In the revision of previous laboratory results, hyponatremia was labeled as chronic since it was consistently present in the previous year. The noted peripheral edema was attributed to hypoalbuminemia since there were no other signs or abnormal markers of volume overload (Table 1). As the patient presented mostly euvolemic, these findings were compatible with syndrome of inappropriate antidiuretic hormone secretion (SIADH). Glucocorticoid deficiency and hypothyroidism were also excluded.

**Figure 1:**
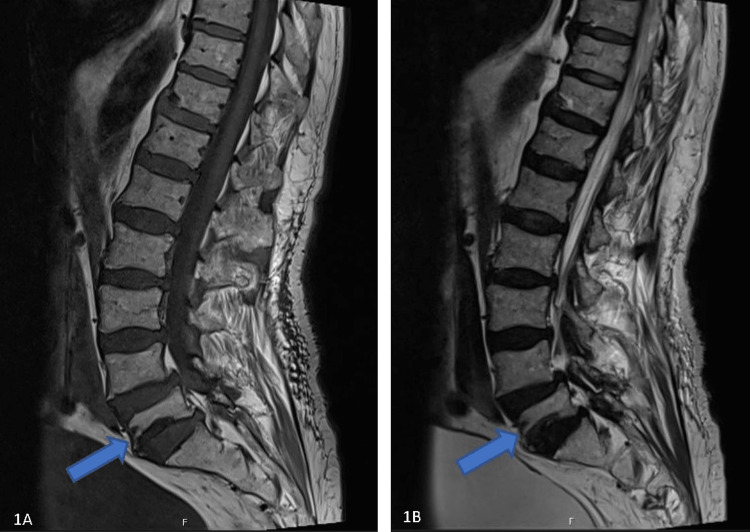
L5 vertebral fracture on T1 (1A) and T2 (1B) weighted lumbosacral MRIs. Blue arrows identify L5 vertebral fracture.

In this setting, a neoplastic lesion (prostate cancer relapse or metastasis in particular) with medullary or neural invasion was the first hypothesis suspected, along with paraneoplastic SIADH. Abdominopelvic, dorsal, and lumbosacral MRIs (with contrast enhancement) were performed and identified L5 vertebral fracture, with no conus medullaris nor medullary compromise elsewhere (Figures 1A, 1B). No signs of neoplastic disease were found.

The serum prostate-specific antigen was below detection range (as it was consistent in all urologic oncology follow-ups after the end of the treatment). Alkaline phosphatase was, initially, slightly increased due to acute bone remodeling (<200 IU/L), and it stabilized in the normal range in further determinations. Head, cervical, thoracic, and abdominal CT scan (as well as referred MRI) excluded vascular or neoplastic lesions. Lumbar fracture was attributed to fall episodes, and conservative treatment was advised by the consulting spine team with lumbar support belt use.

**Figure 2:**
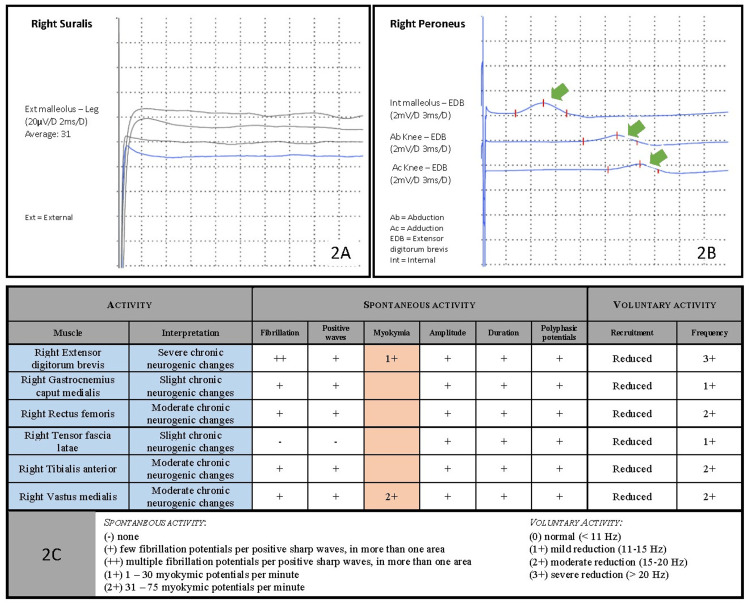
Electromyographic findings showing post-ganglionic sensory-motor impairment with myokymia, supporting lumbosacral plexopathy. The images show (A) absent sensory responses on right suralis, (B) motor nerve reduced amplitudes and delayed motor conduction velocity on right peroneus (green arrows show the delayed waveforms along the right leg), and (C) the final diagram for right leg examination, showing myokymic activity (orange column) and summarizing findings (blue column).

Further investigation with electromyography and evoked potential studies were requested. These examinations revealed post-ganglionic, sensory-motor impairment of both lower limbs, with involuntary slow contractions of superficial muscle fibers - myokymia. Figures 2A-2C show electromyographic activity and summary diagram.

**Figure 3:**
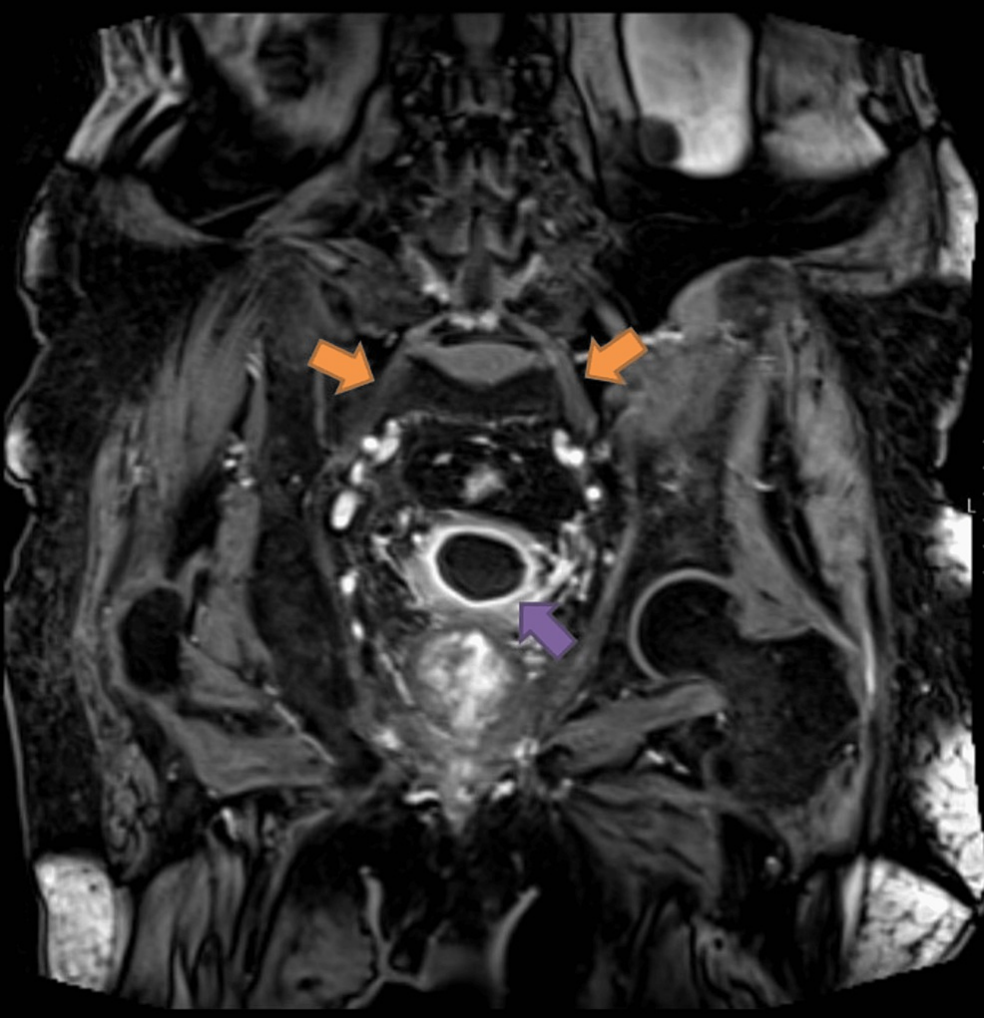
Gadolinium-enhanced T1-weighted coronal image of pelvic MRI (breath-hold volume interpolated body examination with spectral fat saturation sequence {VIBE-FS}). Bilateral lumbosacral plexus thickening with neurogenic edema (orange arrows) and bladder wall thickening with gadolinium diffuse enhancement (purple arrow), supporting lumbosacral plexopathy and radiation-induced cystitis.

These findings were strongly suggestive of a lumbosacral plexus lesion and consistent with the neurological findings. The presence of myokymia was particularly favorable to radiation-induced origin. Specific pelvic MRI with gadolinium enhancement addressing lumbosacral plexus was performed to characterize the extension and the radiological appearance of the lesions. Bilateral thickening of lumbosacral plexus with surrounding pelvic edema was consistent with RILP. Moreover, gadolinium enhancement and thickening of the bladder wall were also favorable to extended radiation-induced cystitis (Figure 3).

Urethral cystoscopy was also requested, showing signs of a congestive bladder with vascular ectasia confirming previous findings of radiation-induced cystitis. These findings explained the hematuria and incontinence previously reported.

Following a multidisciplinary discussion, other causes of plexopathy were excluded, including diabetes (normal glycosylated hemoglobin), infection (negative serologies for HIV, hepatitis B and C virus, cytomegalovirus, Epstein-Barr virus, syphilis and Borrelia spp., and negative interferon-gamma release assay), and autoimmune and neuroimmune screening (including antinuclear, antineuronal and antineutrophil cytoplasmic antibodies). Additionally, serum levels of vitamin B12, folic acid, and complement C3 and C4 were normal, as well as serum creatine kinase and myoglobin.

Lastly, hyponatremia was hard to reverse with the initial measures taken, including dietary protein and sodium reinforcement, introduction of loop diuretic, and water intake restriction. Potential culprit drugs were then suspended or substituted (alfuzosin, amiodarone, and aminophylline) with further normalization of sodium values. Hereby, hyponatremia was associated with drug-induced SIADH.

Under a directed rehabilitation program, the patient was trained to stand and walk with crutches and recovered mobility with partial autonomy. After a six-month follow-up, the patient remained clinically stable, with controlled natremia and no evidence of neoplastic disease.

## Discussion

The diagnosis of RILP is challenging and this case highlights the detailed differential diagnosis. From the clinical perspective, the characterization of pain and neurological defects are key features. In this case, the initial picture had mixed features, but neurological impairment was dominant at presentation, which was more favorable to RILP. In neoplastic LSP, lumbosacral and gluteal pain are main elements reported by 75-91% of patients at presentation [4,6]. Mixed inflammatory and mechanical pattern is common, sometimes with radicular radiation. In a few cases, pain develops in weeks [6]. In almost all cases pain preceded the peripheral neurologic symptoms within weeks to months [6]. Conversely, only a minority of patients with RILP (10-33%) refer pain, usually mild, with a neuropathic pattern, presenting with paresthesia, hyperalgesia, and allodynia [4,6]. As in this case, the presentation of RILP is insidious, with a gradual course, often with progressive flares.

A thorough review of the radiotherapy protocol is important to assess the likelihood of critical exposition. A causal link with late-onset neurologic toxicity is very difficult to establish on a direct basis since the neuronal tolerability profile is not absolute and equal to each individual. As we see in this case, image-guided radiation therapy with on-board imaging (IGRT-OBI) is a very reliable technique in terms of precision of irradiation, standard fractionated doses were administered, and total dose delivered on the prostate gland was according to European Association of Urology and European Society for Radiotherapy and Oncology guidelines [18]. Still, radiation-induced cystitis developed as part of the calculated known risks. Pelvic region was irradiated with total dose of 50 Gy, which is about the critical threshold for increased risk for plexus neurotoxicity. Moreover, known risk factors for RILP like advanced age, hypertension, and obesity could have contributed to the development of the condition.

Electromyography is important not only to establish the level of neurological impairment but also to detect findings supportive of the diagnosis, as we see here with postganglionic pattern and myokymia which is very specific to RILP. Imaging studies are also warranted, and MRI is superior to CT scan in this setting [8,12,16,19]. There are no pathognomonic MRI findings for RILP, but the appearance of smooth longitudinal thickening of fibers with longitudinal thin enhancement is typical of radiation neuritis [19,20]. Metastases have patchy and nodular aspects [12,16,19]. Of note, there is no evidence of neoplastic disease in spine and pelvic MRI, as well as persistent unmeasurable prostate-specific antigen. Spine MRI also identified L5 vertebral fracture, which was related to the recent fall episodes. There were neither conus medullaris compromise, compatible electrophysiological characteristics, nor temporal relations with symptoms. Whether radiation has contributed to bone fragility in this fracture is uncertain.

Another important aspect is to assess clinical evidence of other concomitant radiation-induced toxicity affecting other pelvic organs or bone pelvic structures, as in radiation-induced cystitis, proctitis, avascular bone necrosis, and vertebral stenosis or collapse [1]. The presence of hematuria and urinary incontinence was initially highly suspicious for a neoplastic relapse and euvolemic SIADH raised these concerns. In this setting, cystoscopy and MRI were essential to exclude the latter and document concomitant genitourinary toxicity. Paraneoplastic SIADH was also discarded, and drug-induced SIADH was consistent with the improvement seen with associated drug suspension.

There is no directed treatment for RILP that reverses or prevents its progression [1]. Physical and occupational therapy may help to reduce or recover some autonomy and prevent muscle atrophy [1,8,12]. Patients should avoid local trauma and mechanical stretching of the plexus, which may trigger inflammation and extensive fibrosis. Antidepressants (duloxetine) and GABA-receptor agonists (gabapentin) or analogs (pregabalin) can be used to decrease neuronal hyperexcitability and control neuropathic symptoms. There are no proven benefits of corticosteroid, anticoagulation, or hyperbaric oxygen therapy [8]. The prognosis is uncertain, but long periods of clinical stability may follow presentation. Follow-up of these patients is highly recommended, as well as serial imaging in case of atypical course of the disease or new onset of pain [12,16,17].

## Conclusions

The diagnosis of RILP is challenging and the exclusion of other differential diagnoses is critical, particularly with a neoplastic etiology. History and clinical examination can provide valuable information to support the diagnosis. Radiotherapy protocol must be reviewed to assess the likelihood of harm facing known risk factors. Pelvic and spine MRI imaging and electromyographic studies are also warranted in this setting. Prostate cancer is common in older ages and radiation therapy is more often chosen to treat in situ and localized lesions, instead of major surgery. More robust long-term studies are needed to monitor and characterize RILP and other delayed-onset toxicities, as they might bring severe impairment to the quality of life of cancer survivors.
